# Acetate and propionate vs. iTBS as a novel method for cognitive dysfunction and anxiety symptoms in delayed encephalopathy after acute carbon monoxide poisoning rat

**DOI:** 10.3389/fphar.2025.1520988

**Published:** 2025-02-26

**Authors:** Tianyu Meng, Xin Zhang, Jili Zhao, Hui Xue, Lehua Yu

**Affiliations:** ^1^ Department of Rehabilitation Medicine, The Second Affiliated Hospital of Chongqing Medical University, Chongqing, China; ^2^ Department of Neurology, Baotou Central Hospital, Baotou, Inner Mongolia, China

**Keywords:** DEACMP, iTBS, SCFAs, inflammation, neurotransmitter

## Abstract

**Background:**

The optimal treatment methods for delayed encephalopathy after acute carbon monoxide (CO) poisoning (DEACMP) were not identified. Thus, this study was conducted to compare the efficacies of intermittent theta burst stimulation (iTBS) and short-chain fatty acids (SCFAs) in treating cognitive dysfunction and anxiety symptoms of DEACMP rat.

**Methods:**

In phase I, a DEACMP rat model was built to assess the inflammation levels in the hippocampus and levels of SCFAs in the serum of DEACMP rats. In phase II, DEACMP rats were randomly assigned into four groups: DEACMP + placebo, DEACMP + SCFAs, DEACMP + sham iTBS, and DEACMP + iTBS. The intervention was continued for 2 weeks. A Morris water maze and open field tests were used to assess cognitive function and anxiety symptoms, respectively.

**Results:**

The levels of three inflammatory factors (IL-1β, IL-6, and TNF-α) and two SCFAs (acetate and propionate) were significantly increased and decreased, respectively, in DEACMP rats. After treatment, cognitive dysfunction and anxiety symptoms were significantly improved in the DEACMP + iTBS group and the DEACMP + SCFAs (consisting of acetate and propionate) group. Both SCFAs and iTBS could significantly improve the increased levels of IL-1β, IL-6, and TNF-α in the hippocampus, and SCFAs could also improve the decreased levels of GPR41, GPR43, dopamine, and norepinephrine in the hippocampus of DEACMP rats.

**Conclusion:**

These results indicate that both iTBS and SCFA solutions consisting of acetate and propionate produced good effects on DEACMP rats by regulating inflammation levels in the hippocampus, and acetate/propionate–GPR41/GPR43–IL-1β/IL-6/TNF-α–dopamine/norepinephrine may be a potential pathway in SCFAs for the treatment of DEACMP.

## Introduction

Carbon monoxide (CO) is a difficult-to-detect colorless and odorless gas. It can cause multiple organ dysfunctions, mainly characterized by central nervous system (CNS) damage (Chen et al., 2021; Kinoshita et al., 2020). Delayed encephalopathy after acute CO poisoning (DEACMP) is a common and serious complication after CO poisoning (Xu et al., 2019). Approximately 10%–30% of patients develop DEACMP after CO poisoning, resulting in a great economic burden for individuals and society (Wang et al., 2016). Using a DEACMP rat model, we found that the microglial activation regulated by ATP through the P2Y12 receptor pathway may be closely related to the onset of DEACMP (Xiang et al., 2021), and that inflammation may have an important role in the onset of DEACMP (Xiang et al., 2014). Currently, the pathogenesis of DEACMP is still not fully understood. Patients with DEACMP always experience several severe clinical symptoms, such as dementia and cognitive dysfunction (Harper and Croft-Baker, 2004). Our previous studies reported that the combined application of hyperbaric oxygen and medications, such as dexamethasone (Xiang et al., 2017a) and N-butylphthalide (Xiang et al., 2017b), could be potential new methods to improve cognitive dysfunction in DEACMP patients. However, the optimal treatment methods for DEACMP are still not available. Therefore, it is necessary to further explore novel treatment methods for this disease.

Theta burst stimulation (TBS) is a paradigm of repetitive transcranial magnetic stimulation (rTMS). It can enhance synaptic transmission by mimicking cortical theta rhythms, thus enhancing cortical excitability. Intermittent TBS (iTBS) is one of the two main types of TBS used in clinical research. As a non-invasive brain stimulation method, previous research found that iTBS could be used to treat neuropsychiatric disorders, such as depression and stroke (Scho et al., 2024; Han et al., 2024). More recently, iTBS has emerged as a potential intervention for cognitive dysfunction. Stanojevic et al. found that iTBS could mitigate the cognitive impairment in the streptozotocin-induced model of Alzheimer’s disease (Stanojevic et al., 2022). A recent meta-analysis showed that patients with cognitive dysfunction improved their cognitive function after receiving iTBS (Zheng et al., 2024). These findings indicate that iTBS may be effective in the treatment of DEACMP, although few studies have focused on the efficacy of iTBS DEACMP.

Short-chain fatty acids (SCFAs) are the main products of the gut microbiota and have wide-ranging effects locally and throughout the body. The three major SCFAs are acetate, propionate, and butyrate. They can affect CNS functions by modulating different pathways to the brain, such as the vagus nerve (O'Riordan et al., 2022). SCFAs are now considered to be major communication links between the gut and the brain. Dysfunction of SCFAs is closely linked to many neuropsychiatric disorders, such as depression (Cheng et al., 2024). More and more researchers now use SCFAs as a novel method for the treatment of diseases, such as propionate for multiple sclerosis disease (Duscha et al., 2020). Li et al. reported that SCFAs could significantly improve cognitive dysfunction in mice (Tian et al., 2024). During the processes by which SCFAs exert beneficial effects on host health, two G-protein-coupled receptors (GPRs), GPR41 and GPR43, play important roles and have received increasing attention. GPRs are a diverse class of cell surface receptors that are closely related to many physiological functions, such as the inflammatory response, and GPR41 and GPR43 are the receptors of SCFAs (Kimura et al., 2014). Therefore, we conducted this study to assess whether SCFAs could yield good efficacy in the treatment of DEACMP, and then preliminarily investigate the underlying mechanism of action. Meanwhile, we assessed the efficacy of iTBS on DEACMP and compared its efficacy with that of SCFAs. Our findings can be helpful in exploring the optimal treatment methods for DEACMP.

## Materials and methods

### DEACMP model

Sprague–Dawley rats (6–8 weeks, 180–230 g) were provided by SiPeiFu (Beijing) Biotechnology Co. Ltd. (China) and housed under standard conditions. After 1 week of acclimation, the rats were randomly assigned to either an experimental group or a control group. To build the DEACMP model, according to our previous studies (Xiang et al., 2021; Xiang et al., 2014), the rats in the experimental group received CO (static inhalation, 1,000 ppm/40 min and 3,000 ppm/20 min), and the rats in the control group received the same amount of air. The whole procedure was conducted according to the National Institutes of Health guidelines for animal research (*Guide for the Care and Use of Laboratory Animals*, NIH Publication No.8023, revised 1996). Our study was approved by the Ethics Committee of Baotou Central Hospital.

### Behavioral experiments

In this study, the escape latency in the Morris water maze (MWM) test was used to assess cognitive function, and the center distance as a percentage of total distance (center distance (%)) in the open field test (OFT) was used to assess anxiety symptoms in the rats. These two behavioral experiments were performed exactly according to our previous studies (Xiang et al., 2021; Xiang et al., 2014) to ensure that the control and experimental groups had similar escape latency and center distance (%). In phase I, after 21 days of CO poisoning, these two behavioral experiments were conducted to assess whether the DEACMP model had been successfully built, and then the rats were sacrificed. The hippocampus and serum of the rats were collected to assess the levels of inflammation and the levels of SCFAs, respectively. In phase II, these two behavioral experiments were conducted after 21 days of CO poisoning, after 1 week of treatment, and after 2 weeks of treatment, and then the rats were sacrificed. The hippocampus of the rats was collected to assess inflammation levels and neurotransmitter levels.

### Detection methods

Liquid chromatography-mass spectrometry (LC-MS) was used to detect neurotransmitters in the hippocampus. Briefly, we first weighed the sample and prepared the supernatant (400 μL mixture of methanol-water (4/1, v/v); 2-chloro-l-phenylalanine (75 ng/mL) dissolved in methanol as an internal standard; homogenization at low temperature for 10 min; ultrasonic extraction in an ice water bath for 10 min; centrifugation at 14,000 rpm for 10 min, 4 °C). Then, 300 μL of supernatant and 200 μL of a mixture of methanol-water (4/1, v/v) were transferred to an Eppendorf tube. After vortexing for 30 s, ultrasonic extraction for 5 min, and centrifugation at 14,000 rpm for 10 min at 4 °C, 200 μL of supernatant was transferred to a new Eppendorf tube. In the third step, methanol-water (4/1, v/v) was added to the powder, which was obtained in a freeze-concentration centrifugal dryer. Then, 150 μL of the supernatant obtained after centrifugation (10 min, 14,000 rpm, 4 °C) was transferred to the injection vial for later LC-MS analysis. The quantification of neurotransmitters was conducted on a Waters Acquity UPLC system. In addition, enzyme-linked immunosorbent assay kits (Jianglai Industry Co., Ltd., Shanghai, China) were used to detect interleukin-6 (IL-6), interleukin-1 beta (IL-1β), tumor necrosis factor-α (TNF-α), GPR41, and GPR43. The detection process was according to the manufacturer’s instructions.

### Intervention modalities

**FIGURE 1:**
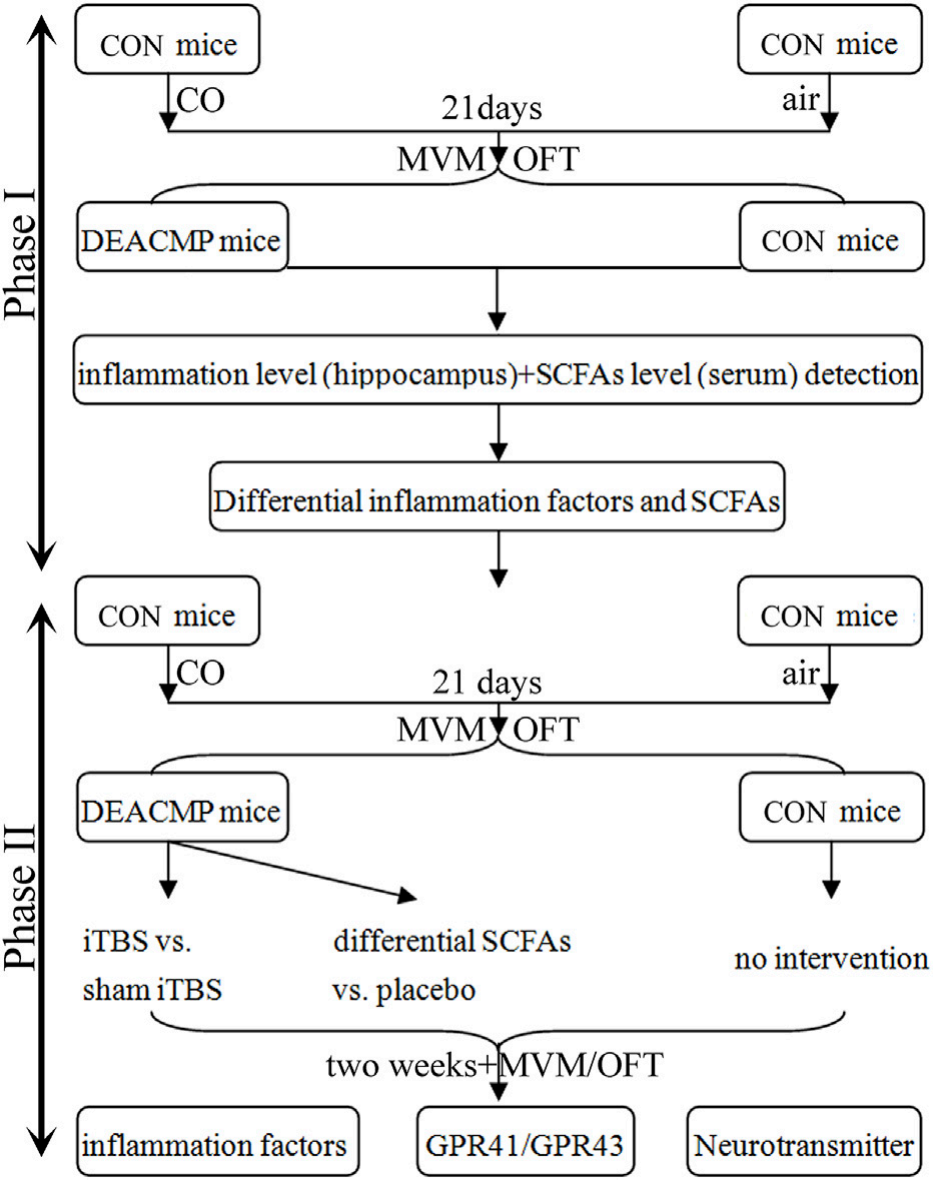
Study design. This study consisted of two phases. Phase I was conducted to identify the differential inflammatory factors in the hippocampus and SCFAs in serum, and phase II explored the efficacy of differential SCFAs in the treatment of DEACMP. iTBS was used as a positive control. CON, control; DEACMP, delayed encephalopathy after acute carbon monoxide poisoning; iTBS, intermittent theta burst stimulation; SCFAs, short-chain fatty acids; MWM, Morris water maze; OFT, open field test; GPR41, G protein-coupled receptor 41; GPR43, G protein-coupled receptor 43.

In phase II, after the DEACMP model was successfully built, the DEACMP rats in the experimental group were randomly assigned into four groups: DEACMP + SCFAs group, DEACMP + placebo group, DEACMP + iTBS group, and DEACMP + sham iTBS group. The SCFA solution consisted of sodium acetate (67.5 mmol/L) and sodium propionate (25.9 mmol/L) in a 3:1 ratio (Tian et al., 2024), and normal saline was used as a placebo. The SCFAs (500 mg/kg per day) and an equal amount of normal saline were given intragastrically (Tian et al., 2024). The iTBS block consisted of twenty trains of ten bursts (three pulses at a frequency of 50 Hz), repeated at 5 Hz (lasting 192 s with 10 s intervals between trains) (Dragic et al., 2020). The MagStim Rapid2 was used to conduct iTBS. The stimulation intensity was set to 30% of the maximum stimulator output, just below the motor threshold. The motor threshold was defined as the stimulation intensity that could cause the minimum visible contraction of the upper limbs (Dragic et al., 2020). In sham iTBS, the coil was rotated 90° around its vertical midline axis to ensure no brain stimulation (Dragic et al., 2020). The intervention was continued for 2 weeks. The setup is described in Figure 1.

### Statistical analysis

SPSS 21.0 and R software 4.2 were used for all the statistical analyses. Student’s t-test was used to find the differences between the two groups. A paired t-test was used here to determine whether the changes between two-time points in one group were significantly different. However, when the data did not meet the prerequisite assumption of normal distribution, non-parametric methods were used to explore the differences between the two groups. Meanwhile, one-way analysis of variance (ANOVA) was used to find the differences among the three groups; if a significant difference was found in this test, Tamhane’s T2 or Bonferroni post-hoc test was then performed to determine which two groups significantly differed according to the equal variance criterion. Spearman’s correlation analysis was used to determine the potential correlations between differential factors. A heat map was used to show the changes in neurotransmitters in the different groups. All tests were two-sided, and p < 0.05 was considered statistically significant.

## Results

### Cognitive dysfunction in DEACMP rats

**FIGURE 2:**
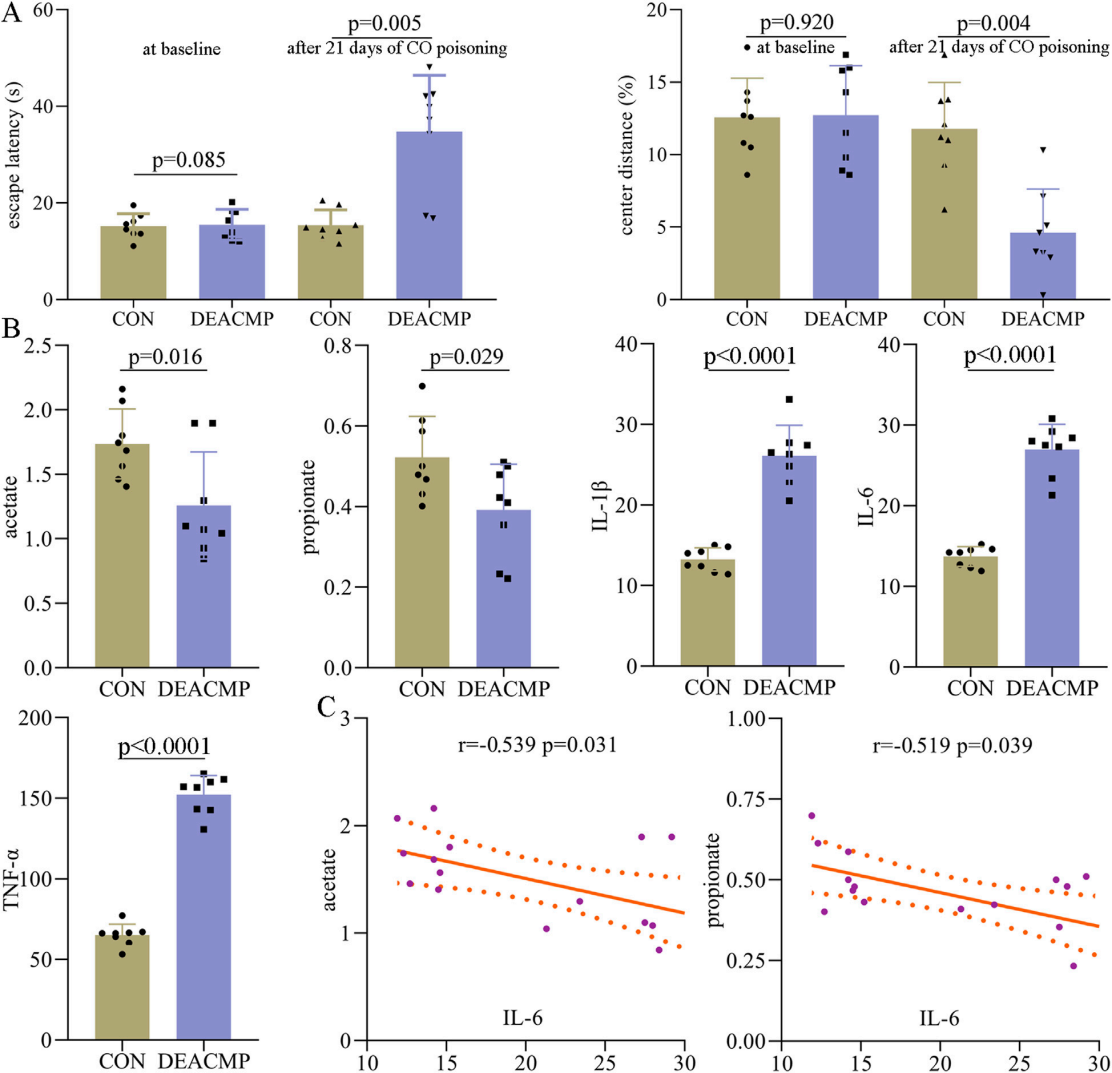
Abnormal behaviors and molecules in DEACMP rats. **(A)** After 21 days of CO poisoning, DEACMP rats showed significantly higher escape latency and lower center distance (%) than control rat. **(B)** Two SCFAs (acetate and propionate) in serum and three inflammatory factors (IL-1β, IL-6, and TNF-α) in the hippocampus were significantly changed in DEACMP rats. **(C)** There were significant negative correlations between IL-6 and these two SCFAs (acetate and propionate). CON, control; DEACMP, delayed encephalopathy after acute carbon monoxide poisoning.

After 21 days of CO poisoning, a DEACMP rat mode was built. To find out whether DEACMP rats had cognitive dysfunction, the Morris water maze test was used. At baseline, the two groups had similar escape latency (p = 0.850), but DEACMP rats had a significantly higher escape latency than control rats (p = 0.0005, Figure 2A). These results indicate that DEACMP rats had cognitive dysfunction. Meanwhile, to explore whether DEACMP rats had anxiety symptoms, an open-field test was used. At baseline, the two groups had similar center distance (%) (p = 0.920), but DEACMP rats eventually had a significantly higher center distance (%) than the control rats (p = 0.0004, Figure 2A). These results show that DEACMP rats had anxiety symptoms.

After being sacrificed, the serum and hippocampus of the rats were collected and stored for later experiments. In this study, eight SCFAs were successfully identified in rat serum: valerate, propionate, isohexanate, isobutyrate, butyrate, acetate, isovalerate, and hexanate. Compared to control rats, DEACMP rats had significantly lower levels of acetate (p = 0.016) and propionate (p = 0.029, Figure 2B). The levels of the other six SCFAs were similar between the two groups: valerate (p = 0.570), isohexanate (p = 0.239), isobutyrate (p = 0.211), butyrate (p = 0.862), isovalerate (p = 0.129), and hexanate (p = 0.644). In addition, we also assessed the inflammation levels in the hippocampus of DEACMP rats. The results showed that compared to control rats, DEACMP rats had significantly higher levels of IL-1β (p < 0.0001), IL-6 (p < 0.0001), and TNF-α (p < 0.0001, Figure 2B). Spearman’s correlation analysis showed that IL-6 was significantly correlated with acetate (r = −0.539, p = 0.031) and propionate (r = −0.519, p = 0.039, Figure 2C). These phase I results demonstrate that DEACMP rats had increased inflammation levels in the hippocampus and decreased levels of SCFAs in serum.

### Efficacy of SCFAs in the treatment of DEACMP rats

**FIGURE 3:**
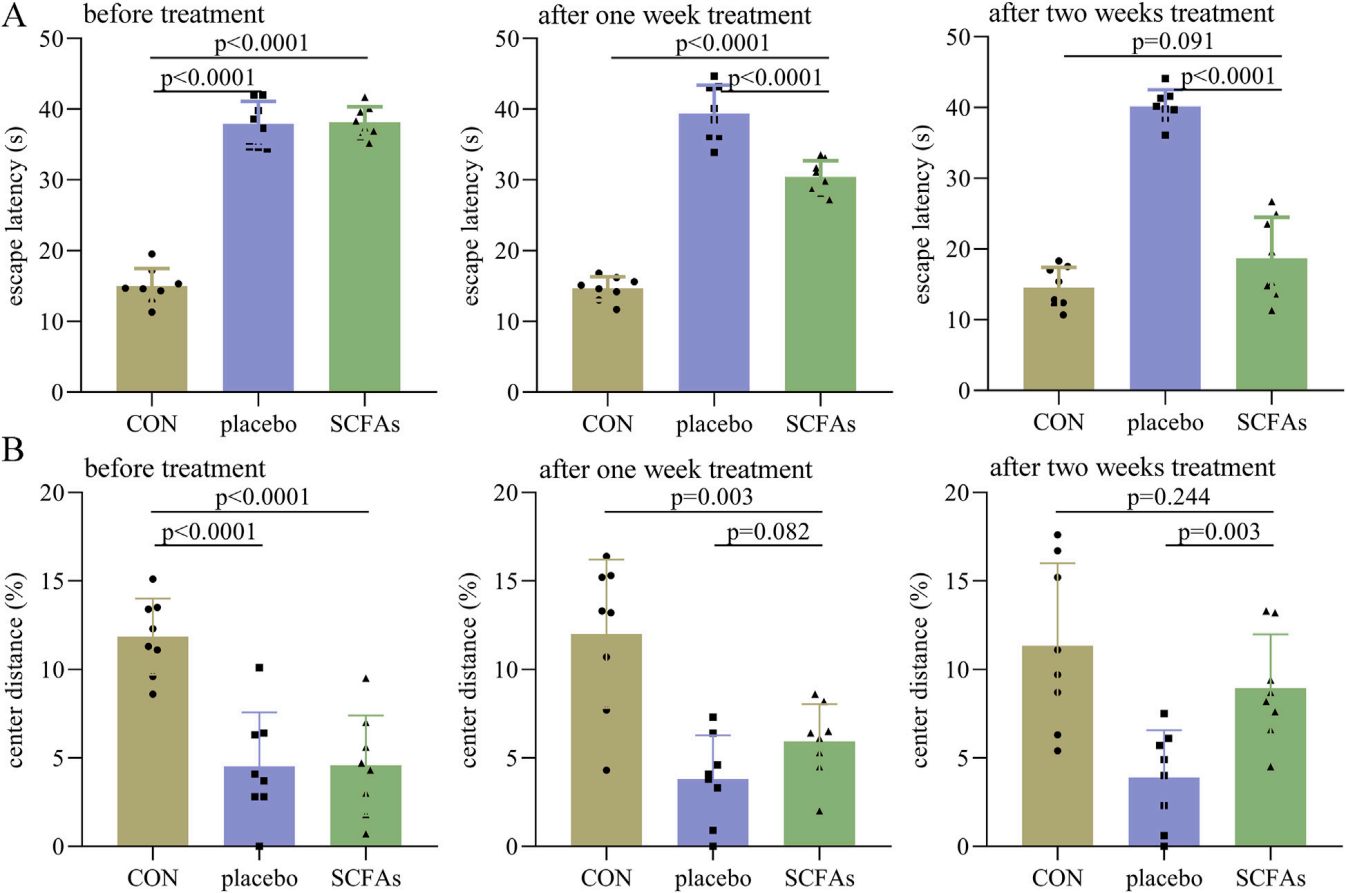
Efficacy of SCFAs on cognitive function and anxiety symptoms in DEACMP rats. **(A)** Before treatment, the DEACMP + placebo group and the DEACMP + SCFAs group had similar escape latency. After treatment, the DEACMP + SCFAs group had a significantly lower escape latency than the DEACMP + placebo group. **(B)** Before treatment, the DEACMP + placebo group and the DEACMP + SCFAs group had a similar center distance (%). After 1 week of treatment, the DEACMP + SCFAs group had a non-significantly higher center distance (%) than the DEACMP + placebo group; after 2 weeks of treatment, the DEACMP + SCFAs group had a significantly higher center distance (%) than the DEACMP + placebo group. CON, control; DEACMP, delayed encephalopathy after acute carbon monoxide poisoning; SCFAs, short-chain fatty acids.

In phase II, before treatment, the control group had a significantly lower escape latency than both the DEACMP + placebo group (p < 0.0001) and the DEACAMP + SCFAs group (p < 0.0001, Figure 3A). After 1 week of treatment, the escape latency in the DEACAMP + SCFAs group was significantly lower than that of the DEACMP + placebo group (p < 0.0001), but still significantly higher than the control group (p < 0.0001, Figure 3A). After 2-weeks of treatment, the escape latency in the DEACMP + SCFAs group was significantly lower than that in the DEACMP + placebo group (p < 0.0001) and similar to that in the control group (p = 0.091, Figure 3A). Meanwhile, we found that, in the DEACMP + SCFAs group, the escape latency after 2 weeks of treatment was significantly lower than the escape latency after 1 week of treatment (p = 0.0001), and the latter was significantly lower than the escape latency after 21 days of CO poisoning (p < 0.0001). Similar results were found for the center distance (%) after SCFA treatment (Figure 3B). These results indicate that SCFAs could significantly improve cognitive dysfunction and anxiety symptoms in DEACMP rats.

### Efficacy of iTBS in the treatment of DEACMP rats

**FIGURE 4:**
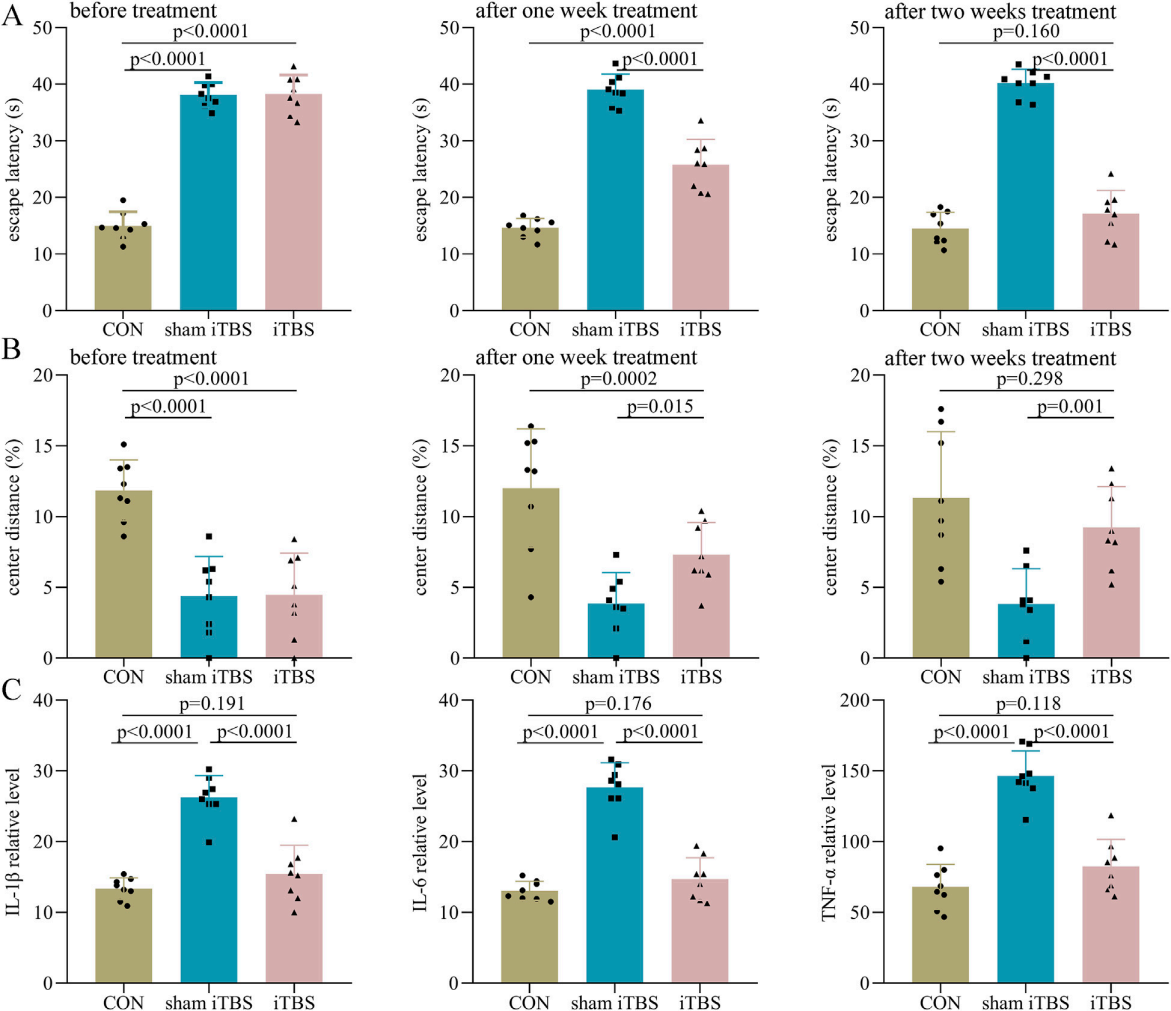
Efficacy of iTBS on cognitive function and anxiety symptoms in DEACMP rats. **(A)** Before treatment, the DEACMP + sham iTBS group and the DEACMP + iTBS group had similar escape latency. After treatment, the DEACMP + iTBS group had a significantly lower escape latency than the DEACMP + sham iTBS group. **(B)** Before treatment, the DEACMP + sham iTBS group and DEACMP + iTBS groups had similar escape latency. After treatment, the DEACMP + iTBS group had a significantly higher center distance (%) than the DEACMP + sham iTBS group. **(C)** After 2 weeks of treatment, IL-1β, IL-6, and TNF-α levels were significantly lower in the DEACMP + iTBS group than in the DEACMP + sham iTBS group, but their levels were similar between the CON and the DEACMP + iTBS groups. CON, control; DEACMP, delayed encephalopathy after acute carbon monoxide poisoning; iTBS, intermittent theta burst stimulation.

In phase II, before treatment, the control group had a significantly lower escape latency than both the sham iTBS (p < 0.0001) and iTBS (p < 0.0001) groups (Figure 4A). After 1 week of treatment, the escape latency in the iTBS group was significantly lower than in the sham iTBS group (p < 0.0001), but still significantly higher than in the control group (p < 0.0001, Figure 4A). After 2 weeks of treatment, the escape latency in the iTBS group was significantly lower than that in the sham iTBS group (p < 0.0001), and similar to that in the control group (p = 0.160, Figure 4A). Meanwhile, we found that in the iTBS group, the escape latency after 2 weeks of treatment was significantly lower than the escape latency after 1 week of treatment (p = 0.001). Similar results were found for the center distance (%) after iTBS treatment (Figure 4B). Meanwhile, at the end of the intervention, we found that the levels of IL-1β (p < 0.0001), IL-6 (p < 0.0001), and TNF-α (p < 0.0001) were significantly lower in the iTBS group than in the sham iTBS group, but the levels of these inflammatory factors were similar between the control and iTBS groups (Figure 4C). These results indicate that iTBS could significantly improve cognitive dysfunction and anxiety symptoms in DEACMP rats and restore the increased inflammation levels in the hippocampus of DEACMP rats.

### SCFAs vs. iTBS in the treatment of DEACMP rats

**FIGURE 5:**
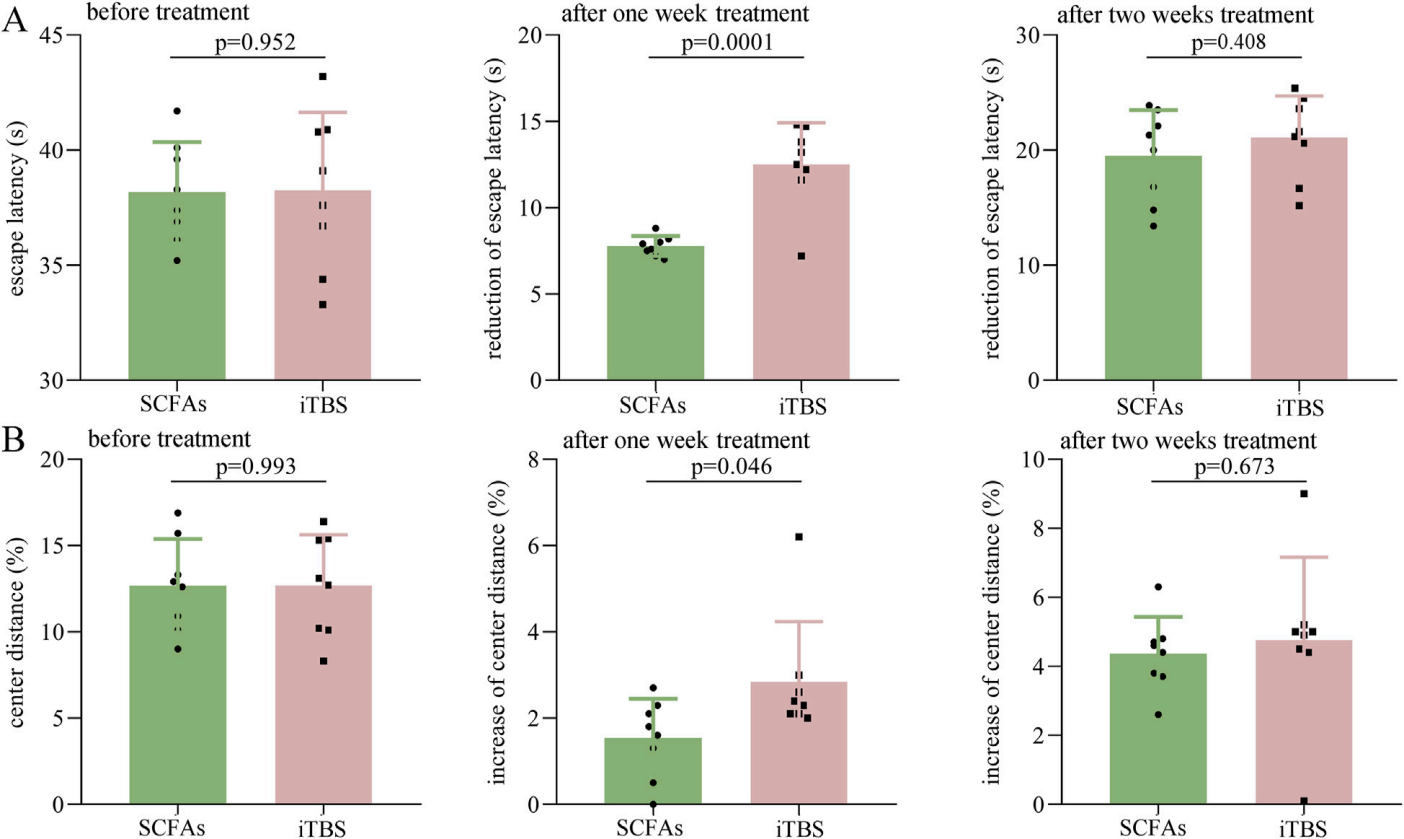
Efficacy of SCFAs versus iTBS in the treatment of DEACMP rats. **(A)** Before treatment, the DEACMP + SCFAs and DEACMP + iTBS groups had similar escape latency. The reduction in escape latency was significantly higher in the DEACMP + iTBS group than in the DEACMP + SCFAs group after 1 week of treatment but was similar between the two groups after 2 weeks of treatment. **(B)** Before treatment, the two groups had similar center distance (%). The increase in center distance (%) was significantly higher in the DEACMP + iTBS group than in the DEACMP + SCFAs group after 1 week of treatment but was similar between the two groups after 2 weeks of treatment. DEACMP, delayed encephalopathy after acute carbon monoxide poisoning; SCFAs, short-chain fatty acids; iTBS, intermittent theta burst stimulation.

To explore whether these two different treatment methods have different effects in the treatment of DEACMP rats, the escape latency and center distance (%) between the DEACMP + SCFAs and the iTBS groups were compared. As shown in Figure 5A, the two groups had a similar escape latency before treatment (p = 0.952). After 1 week of treatment, the reduction in escape latency was significantly higher in the iTBS group than in the SCFA group (p = 0.0001); however, after 2 weeks of treatment, the two groups had a similar reduction in escape latency (p = 0.408) (Figure 5A). As shown in Figure 5B, the two groups had similar center distance (%) before treatment (p = 0.993). After 1 week of treatment, the increase in center distance (%) was significantly higher in the iTBS group than in the SCFAs group (p = 0.046), but after 2 weeks of treatment, the two groups had a similar reduction in escape latency (p = 0.673) (Figure 5B). These results suggest that iTBS may work faster in the treatment of cognitive dysfunction and anxiety symptoms in DEACMP rats.

### Inflammatory levels after SCFA treatment

**FIGURE 6:**
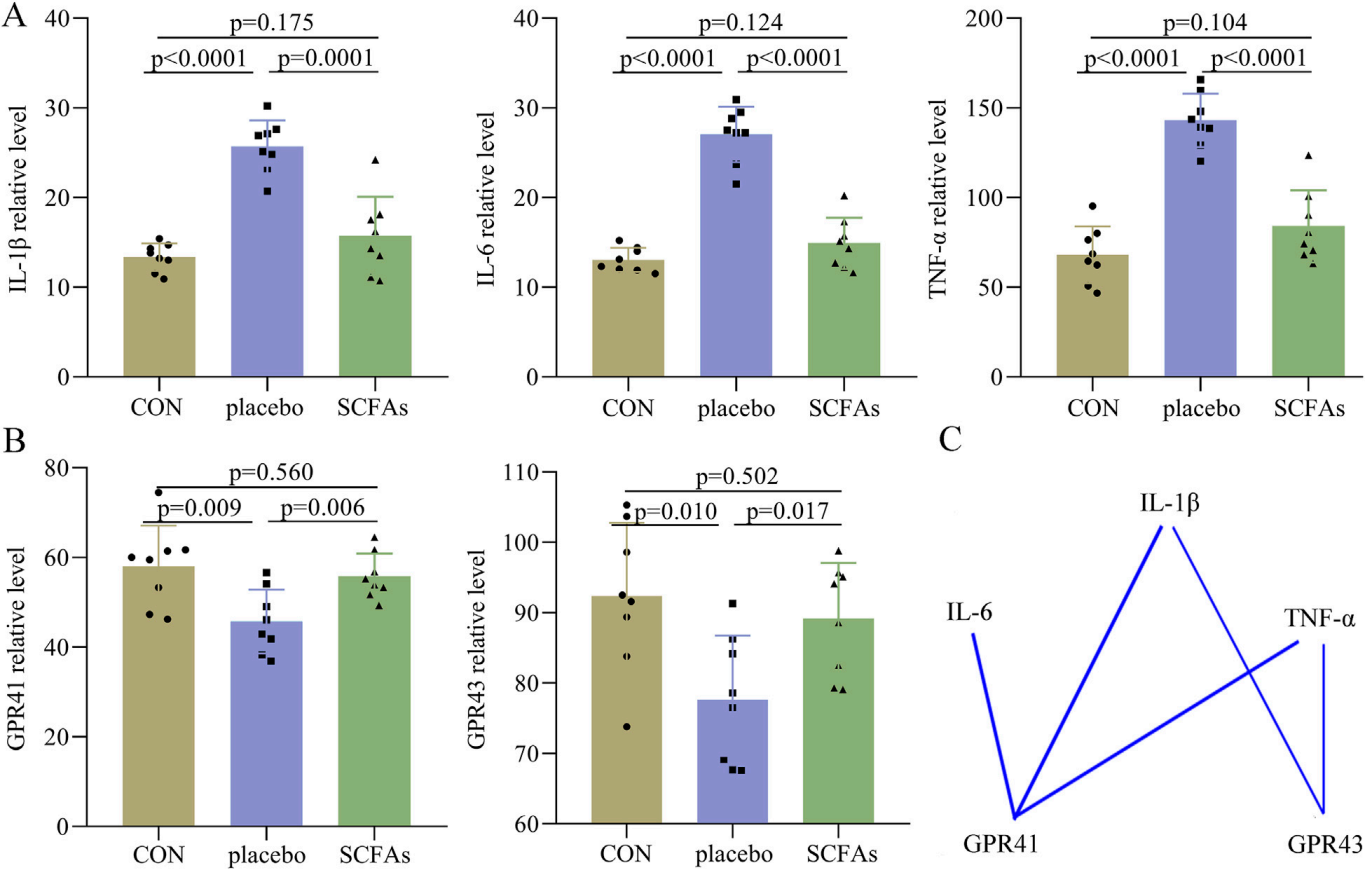
Effects of SCFAs on inflammatory factors and SCFA receptors. **(A)** At the end of treatment, we found that the DEACMP + placebo group had significantly higher levels of IL-1β, IL-6, and TNF-α than the control group, but the levels of these inflammatory factors were similar between the control group and DEACMP + SCFA group. **(B)** At the end of treatment, we found that the DEACMP + placebo group had significantly lower levels of GPR41 and GPR43 than the control group, but the levels of these inflammatory factors were similar between the control group and the DEACMP + SCFAs group. **(C)** There were significant correlations between IL-1β/IL-6/TNF-α and GPR41/GPR43. The blue line indicates a negative correlation. CON, control; DEACMP, delayed encephalopathy after acute carbon monoxide poisoning; iTBS, intermittent theta burst stimulation; GPR41, G protein-coupled receptor 41; GPR43, G protein-coupled receptor 43.

At the end of the intervention (Figure 6A), compared to the control, the DEACMP + placebo group had significantly higher levels of IL-1β (p < 0.0001), IL-6 (p < 0.0001), and TNF-α (p < 0.0001). However, we found that the levels of IL-1β (p = 0.175), IL-6 (p = 0.124), and TNF-α (p = 0.104) were similar between the control and the DEACMP + SCFA groups; the levels of IL-1β (p = 0.0001), IL-6 (p < 0.0001), and TNF-α (p < 0.0001) were significantly lower in the DEACMP + SCFA group than in DEACMP + placebo group. These results showed that SCFAs could significantly relieve inflammation levels in the hippocampus of DEACMP rats.

Both GPR41 and GPR43 levels were found to be significantly lower in the DEACMP + placebo group than in both the control (p = 0.009, p = 0.006) and DEACMP + SCFAs groups (p = 0.010, p = 0.017, Figure 6B). The control and DEACMP + SCFAs groups had similar GPR41 (p = 0.560) and GPR43 (p = 0.502) levels (Figure 6B). Spearman’s correlation analysis was used to determine the potential correlations between IL-1β/IL-6/TNF-α and GPR41/GPR43. The results showed that GPR41 was significantly correlated with IL-1β (r = −0.626, p = 0.001), IL-6 (r = −0.620, p = 0.001), and TNF-α (r = −0.538, p = 0.007), and GPR43 was significantly correlated with IL-1β (r = −0.416, p = 0.043) and TNF-α (r = −0.422, p = 0.040, Figure 6C). These results show that SCFAs may regulate inflammation levels through their receptors, such as GPR41 and GPR43.

### Neurotransmitter levels after SCFA treatment

**FIGURE 7:**
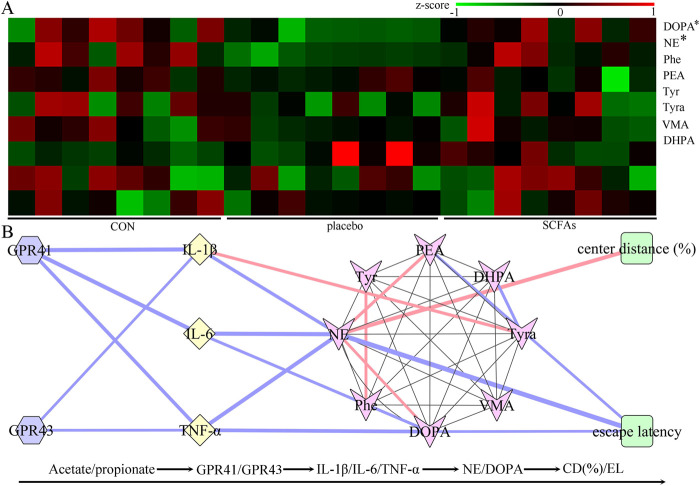
Potential pathway of SCFAs in the treatment of DEACMP. **(A)** After 2 weeks of SCFA treatment, the decreased levels of dopamine and norepinephrine in DEACMP rats were significantly increased. **(B)** There were significant correlations between the differential inflammatory factors and catecholamine neurotransmitters (mainly dopamine and norepinephrine), and escape latency and center distance (%) were also mainly correlated with dopamine and norepinephrine. Considering the relationships between SCFA receptors (GPR41 and GPR43) and inflammatory factors, these results suggest that Acetate/propionate–GPR41/GPR43–IL-1β/IL-6/TNF-α–dopamine/norepinephrine may be a potential SCFA pathway in the treatment of DEACMP. Blue and pink lines indicate negative and positive correlations, respectively; the thicker the line, the greater the correlation coefficient. The * indicates the p-value of the DEACMP + SCFA group vs. the DEACMP + placebo group (DOPA, p = 0.002; NE, p = 0.004). CON, control; DEACMP, delayed encephalopathy after acute carbon monoxide poisoning; SCFAs, short-chain fatty acids; Phe, L-phenylalanine; PEA, phenylethylamine; Tyr, L-tyrosine; Trya, tyramine; DOPA, dopamine; NE, norepinephrine; DHPA, 3,4-dihydroxyphenylacetic acid; VMA, vanillylmandelic acid; GPR41, G protein-coupled receptor 41; GPR43, G protein-coupled receptor 43.

Neurotransmitters in the catecholamine pathway were detected in this study, and eight neurotransmitters in the hippocampus were successfully identified: dopamine, L-phenylalanine, vanillylmandelic acid, phenylethylamine, tyramine, 3,4-dihydroxyphenylacetic acid, L-tyrosine, and norepinephrine (Figure 7A). Compared to the control group, the DEACMP + placebo group had significantly lower levels of dopamine (p = 0.008) and norepinephrine (p = 0.003). However, compared to the control group, the DEACMP + SCFAs group had similar levels of dopamine (p = 0.861) and norepinephrine (p = 0.589). The DEACMP + SCFAs group had significantly higher levels of dopamine (p = 0.002) and norepinephrine (p = 0.004) than the DEACMP + placebo group. These results indicate that SCFAs could effectively improve dopamine and norepinephrine dysregulations.

Spearman’s correlation analysis was used to investigate the potential correlations between differential neurotransmitters and inflammatory factors. The results revealed close relationships between the differential neurotransmitters and inflammatory factors: dopamine vs. IL-6 (r = −0.454, p = 0.026) and TNF-α (r = −0.568, p = 0.004); norepinephrine vs. IL-1β (r = −0.513, p = 0.010), IL-6 (r = −0.633, p = 0.0010), and TNF-α (r = −0.600, p = 0.002, Figure 7B). We found that the escape latency was significantly correlated with dopamine (r = −0.410, p = 0.046) and norepinephrine (r = −0.733, p < 0.0001), and center distance (%) was significantly correlated with norepinephrine (r = 0.603, p = 0.002, Figure 7B)). These results indicate that these two differential neurotransmitters may play important roles in inflammation levels and behavior in DEACMP rats.

## Discussion

In phase I of this study, we found that DEACMP rats had significant cognitive dysfunction and anxiety symptoms compared to control rats. The significantly increased levels of three inflammatory factors (IL-1β, IL-6, and TNF-α) in the hippocampus and decreased levels of two SCFAs (acetate and propionate) in the serum were found in the DEACMP rats. In phase II of this study, we found that both SCFA solution (acetate and propionate) and iTBS could effectively relieve cognitive dysfunction and anxiety symptoms in DEACMP rats. After 2 weeks of treatment, the decreased SCFA receptors (GPR41 and GPR43), increased inflammatory factors (IL-1β, IL-6, and TNF-α), and decreased neurotransmitters (dopamine and norepinephrine) were significantly improved in DEACMP + SCFAs rats. These results suggested that the cognitive dysfunction and anxiety symptoms may be closely related to the inflammation or neurotransmitter levels in the hippocampus of DEACMP rats, and the combination of acetate and propionate may be an effective method for DEACMP. Acetate/propionate–GPR41/GPR43–IL-1β/IL-6/TNF-α–dopamine/norepinephrine may be a potential pathway in SCFA treatment for DEACMP.

rTMS can have neuroprotective effects. Compared to rTMS, iTBS—as a new model of rTMS—needs a shorter time and can produce more long-lasting effects (Chung et al., 2015). However, the neuroprotective mechanisms of iTBS are still unknown. Current evidence suggests that the mechanism of action of this method is mainly related to the regulation of neuronal excitability (Pabst et al., 2022). Shen et al. (2024) reported that iTBS played a neuroprotective role in cerebral ischemia/reperfusion injury by inhibiting endoplasmic reticulum stress and ferroptosis. Considering the close interactions between the nervous and immune systems (Yoo and Mazmanian, 2017), we speculated that iTBS could reduce neuronal death by affecting the immune system and then exerting neuroprotective effects. Interestingly, our study found that iTBS could significantly improve cognitive dysfunction and inflammation levels in DEACMP rats. However, due to the lack of direct evidence, this speculation must be explored in future studies.

SCFAs have a crucial role in maintaining immune functions, host metabolism, and the gut barrier (Kim, 2023). As a two-carbon SCFA, acetate not only serves as an important energy source but also has an anti-inflammatory function. Previous research found that acetate could regulate the nucleotide-binding oligomerization domain-like receptor protein 3 (NLRP3) inflammasome via GPR43 and Ca2+-dependent mechanisms (Jiang et al., 2019). Propionate, a three-carbon SCFA, can also reduce inflammation levels in the body. Filippone et al. (2020) found that propionate has anti-inflammatory properties and proposed that it could be a potential treatment method for inflammatory diseases. In this study, we found that compared to DECAMP + placebo rats, DECAMP + SCFA rats had significantly lower levels of IL-1β, IL-6, and TNF-α in the hippocampus. Moreover, the levels of GPR41 and GPR43 were significantly increased in DECAMP + SCFAs rats. Considering that GPR41 and GPR43 are the important receptors in microglia (Butovsky and Weiner, 2018), our present results suggest that the combination of acetate and propionate produced anti-inflammatory effects by increasing GPR41 and GPR43 levels to regulate the state of microglia which ultimately improved the cognitive dysfunction and anxiety symptoms in DEACMP rats.

Catecholamines have the closest relationships with many CNS functions, such as cognitive function and motor control (Kobayashi, 2001). Disruptions in the catecholamine pathway are involved in many neuropsychiatric diseases. Currently, dopamine is known to play a crucial role in most cognitive functions, and dopamine dysregulation is viewed as a hallmark of many neuropsychiatric diseases (Costa and Schoenbaum, 2022). Norepinephrine is synthesized from dopamine and has been found to be related to cognitive deficits in maternal mice (Liu et al., 2020). In this study, we found two significantly changed catecholaminergic neurotransmitters (dopamine acid and norepinephrine) in DEACMP rats, and their levels were significantly restored after SCFA intervention. In addition, increased inflammation levels in the brain may disrupt the neurotransmitters there (Gorji, 2022; Oshaghi et al., 2023). Therefore, the results of our study indicated that SCFAs might regulate the catecholamine pathway by modulating the inflammation levels in the hippocampus to ultimately produce good effects in DEACMP rats.

Additionally, previous research has reported a close relationship between SCFAs and neurogenesis (Vicentini et al., 2021; Agnihotri and Mohajeri, 2022). Neurogenesis is the process of brain cell growth and repair. Vicentini et al. (2021) found that SCFA could regulate enteric neuronal survival and also stimulate neurogenesis. Agnihotri and Mohajeri (2022) found that SCFAs had a direct effect on neuronal growth and neurogenesisin the hippocampus. Interestingly, Ochi et al. (2021) reported an association between delayed CO encephalopathy and the impairment of adult neurogenesis. These results indicate that SCFAs may produce effects on DEACMP by regulating neurogenesis. Meanwhile, evidence has shown neurogenesis to be influenced by neurotransmitters (Ojeda and Ávila, 2019; Cameron et al., 1998; Berg et al., 2013). In this study, we found that the dopamine and norepinephrine disruptions in the hippocampus of DEACMP rats were significantly improved after SCFA treatment. These findings suggest that the efficacy of SCFAs in the treatment of DEACMP may be related to their potential effects on neurogenesis.

Several limitations should be mentioned here. i) The comparative efficacy of iTBS and SCFAs was assessed with 2-week treatment durations. Thus, the long-term efficacy of these two treatment methods was not assessed here. ii) Only one kind of acetate/propionate dose was used in this study. Future studies should be conducted to assess the efficacy of different acetate/propionate doses in the treatment of DEACMP. iii) In phase I, we found that only acetate and propionate were significantly changed in DEACMP rats; thus, in phase II, only these two SCFAs were used to treat DEACMP rats. However, considering that butyrate was also one of the main three SCFAs, future studies should further explore the efficacy of acetate/propionate/butyrate in the treatment of DEACMP.

## Conclusion

Our study first investigated the efficacy of iTBS and SCFAs in DEACMP rats. The increased inflammation levels in the hippocampus of DEACMP rats could be restored after iTBS or SCFA treatment, and SCFAs could also improve the decreased levels of dopamine and norepinephrine in the hippocampus of DEACMP rats. We also found that iTBS might work faster in improving cognitive dysfunction than SCFAs; however, considering the potential side effects and tolerability of iTBS, SCFAs might be more appropriate for DEACMP. However, our findings can be used in future studies for validation and support.

## Data Availability

The original contributions presented in the study are included in the article/supplementary material; further inquiries can be directed to the corresponding authors.
